# SFAlab: image-based quantification of mechano-active ventral actin stress fibers in adherent cells

**DOI:** 10.3389/fcell.2023.1267822

**Published:** 2023-09-15

**Authors:** Dylan Mostert, Janine Grolleman, Mark C. van Turnhout, Bart G. W. Groenen, Vito Conte, Cecilia M. Sahlgren, Nicholas A. Kurniawan, Carlijn V. C. Bouten

**Affiliations:** ^1^ Department of Biomedical Engineering, Laboratory for Cell and Tissue Engineering, Eindhoven University of Technology, Eindhoven, Netherlands; ^2^ Institute for Complex Molecular Systems (ICMS), Eindhoven University of Technology, Eindhoven, Netherlands; ^3^ Faculty of Science and Engineering, Åbo Akademi University, Turku, Finland

**Keywords:** ventral stress fibers, mechanobiology, microscopy, quantitative image analysis, adherent cells

## Abstract

Ventral actin stress fibers (SFs) are a subset of actin SFs that begin and terminate at focal adhesion (FA) complexes. Ventral SFs can transmit forces from and to the extracellular matrix and serve as a prominent mechanosensing and mechanotransduction machinery for cells. Therefore, quantitative analysis of ventral SFs can lead to deeper understanding of the dynamic mechanical interplay between cells and their extracellular matrix (mechanoreciprocity). However, the dynamic nature and organization of ventral SFs challenge their quantification, and current quantification tools mainly focus on all SFs present in cells and cannot discriminate between subsets. Here we present an image analysis-based computational toolbox, called SFAlab, to quantify the number of ventral SFs and the number of ventral SFs per FA, and provide spatial information about the locations of the identified ventral SFs. SFAlab is built as an all-in-one toolbox that besides analyzing ventral SFs also enables the identification and quantification of (the shape descriptors of) nuclei, cells, and FAs. We validated SFAlab for the quantification of ventral SFs in human fetal cardiac fibroblasts and demonstrated that SFAlab analysis i) yields accurate ventral SF detection in the presence of image imperfections often found in typical fluorescence microscopy images, and ii) is robust against user subjectivity and potential experimental artifacts. To demonstrate the usefulness of SFAlab in mechanobiology research, we modulated actin polymerization and showed that inhibition of Rho kinase led to a significant decrease in ventral SF formation and the number of ventral SFs per FA, shedding light on the importance of the RhoA pathway specifically in ventral SF formation. We present SFAlab as a powerful open source, easy to use image-based analytical tool to increase our understanding of mechanoreciprocity in adherent cells.

## 1 Introduction

To establish and maintain mechanical homeostasis, adherent cells adapt their morphological and mechanical state in response to mechanical cues from their local microenvironment (Vogel and Sheetz, 2006). It has become evident that cell behavior is regulated by mechanical cues from their environment (Vogel and Sheetz, 2006; Van Helvert et al., 2018), and that the regulation of mechanical homeostasis relies on the dynamic mechanical interplay between cells and their extracellular matrix (ECM), a phenomenon referred to as mechanoreciprocity (Van Helvert et al., 2018; De Luca et al., 2021). A myriad of proteins is involved in mechanoreciprocity, starting with the ECM-connecting integrins. Integrins are transmembrane proteins that recognize specific ECM ligands at the extracellular domain, and connect to the actin cytoskeleton at the intracellular domain via a collection of linker proteins (Jansen et al., 2017). A collective of integrins and associated linker proteins is called a focal adhesion (FA), and the assembly, disassembly, and maturation of the FAs is regulated by the forces exerted on the FAs (Kuo, 2013). For mechanoreciprocity, talin and vinculin are important linker proteins in FAs, connecting the integrins to the contractile actomyosin machinery and transmitting force from the ECM to the actomyosin network and *vice versa* (Jansen et al., 2017).

The actomyosin machinery in cells functions as a mechanical stress generator and transducer to enable various processes, such as tissue morphogenesis, cell migration, and muscle contraction (Murrell et al., 2015). In brief, myosin II motors bind to filamentous actin (F-actin) polymers and drive the translocation of two bound F-actin polymers, resulting in a contraction or extension force (Murrell et al., 2015). The assembly of the actomyosin machinery consists of two important steps: 1) establishment of FA sites, and 2) recruitment of F-actin and associated proteins into contractile bundles, which is mediated by the Rho signaling pathway (Small et al., 1998).

Myosin II and F-actin are held together by crosslinking proteins and together form complex structures that are called stress fibers (SFs). Based on the FA connectivity and structural organization, different subtypes of SFs are defined (e.g., dorsal SFs, ventral SFs, transverse arcs, and perinuclear actin). Ventral SFs are generally accepted to be particularly relevant in mechanoreciprocity, because they are connected to the ECM *via* FAs at both ends and consist of contractile actomyosin bundles rich in myosin II, although complete understanding of their role is missing (Tojkander et al., 2012). Ventral SFs have different lengths and orientations within the cell and are mostly found at the ventral surface of a cell, believed to play an important role in cell migration (Narasimhan, 2022). Since ventral SFs are under constant tension, they are assumed to be almost straight filaments without significant buckling (Narasimhan, 2022). To date, our understanding of ventral SF behavior mainly evolved from qualitative studies, demonstrating the complex nature of these structures, and urging the need to accurately quantify them in space and time to unravel their role in mechanosensing and mechanoresponsive.

In contrast to the easily distinguishable FAs, the organization and dynamics of SFs challenge their identification and subsequent quantification via analysis of microscopic images. So far, several image analysis-based tools have been developed to quantify SFs using fluorescent imaging (Rogge et al., 2017; Zhang et al., 2017; Zonderland et al., 2019; Nowak et al., 2020). While most tools aim to quantify all SFs present in cells, very few diversify towards the identification and quantification of individual SF subsets, such as the ventral SFs (Elosegui-Artola et al., 2014; Hauke et al., 2021). For the quantification of ventral SFs, Hauke et al. identified FAs and SFs in cells from microscopic images, and cross-correlated their positions to assess, amongst others, the number of ventral SFs per cell and ventral SF length (Hauke et al., 2021). While this method results in fast coupled analysis of FAs and SFs, the output is dependent on the accuracy and robustness of the correlation procedure, and on user subjectivity. Another approach, published by Elosegui-Artola et al., utilized the location of the FAs as starting and end points for a curve fitting procedure to identify ventral SFs based on maximum intensity projections, thus avoiding the need for cross-correlation (Elosegui-Artola et al., 2014). This method allows identification of curved SFs but has the drawback that the skew of such curves is determined by the orientation of the SF in relation to the reference image. Moreover, this method does not allow for manual disregard of identified curves that do not represent ventral SFs.

In this study, we present an easy-to-use, freely available image analysis-based algorithm, called SFAlab, that circumvents the drawbacks of orientation dependency and the lack of user control over omitting the identification of ventral SFs that are biologically unreasonable. Starting from a similar approach as Elosegui-Artola et al., SFAlab identifies ventral SFs in fluorescent images of cells in 2 dimensions (2D) stained for vinculin and F-actin, based on their maximum intensity. To prevent the fitting of curves that do not represent ventral SFs, we constructed a penalty function in the fitting algorithm that discourages SFAlab to fit curves with shapes that are unrealistic for ventral SFs (e.g., too much curvature or too large relative length). Moreover, our algorithm can fit curves of arbitrary orientation, which enables comparison of the same ventral SFs over time.

We first validate SFAlab and show that the algorithm can be used to determine the number of SFs and the amount of SFs/FA for human cardiac fibroblasts (cFBs), together with the maximum curvature and relative length of the ventral SFs found. Next, we demonstrate the accuracy and robustness of SFAlab in the presence of typically occurring image imperfections and user subjectivity. Finally, we show the biological relevance of ventral SF detection using SFAlab. We demonstrate that we can quantify the influence of Y-27632, a highly selective inhibitor of the RhoA pathway that promotes SF assembly (Iwamoto et al., 2000; Darenfed et al., 2007), on ventral SF formation, showing the direct effect of the RhoA pathway on the formation of ventral SFs in cFBs. Together, our findings indicate SFAlab to be a powerful tool to identify and quantify ventral SFs, which can progress our understanding of the mechanisms behind ventral SF formation and the role of ventral SFs in the dynamic mechanical reciprocity of adherent cells *in vitro*.

## 2 Materials and methods

### 2.1 Code availability

The SFAlab source code has been written in MATLAB (version 8.6.0.267,246, R2015b, glnax64) and can be found, together with a detailed user manual, on https://gitlab.tue.nl/stem/sfalab. SFAlab is open-access and freely available for use and further development.

### 2.2 SFAlab development and working principle

**FIGURE 1:**
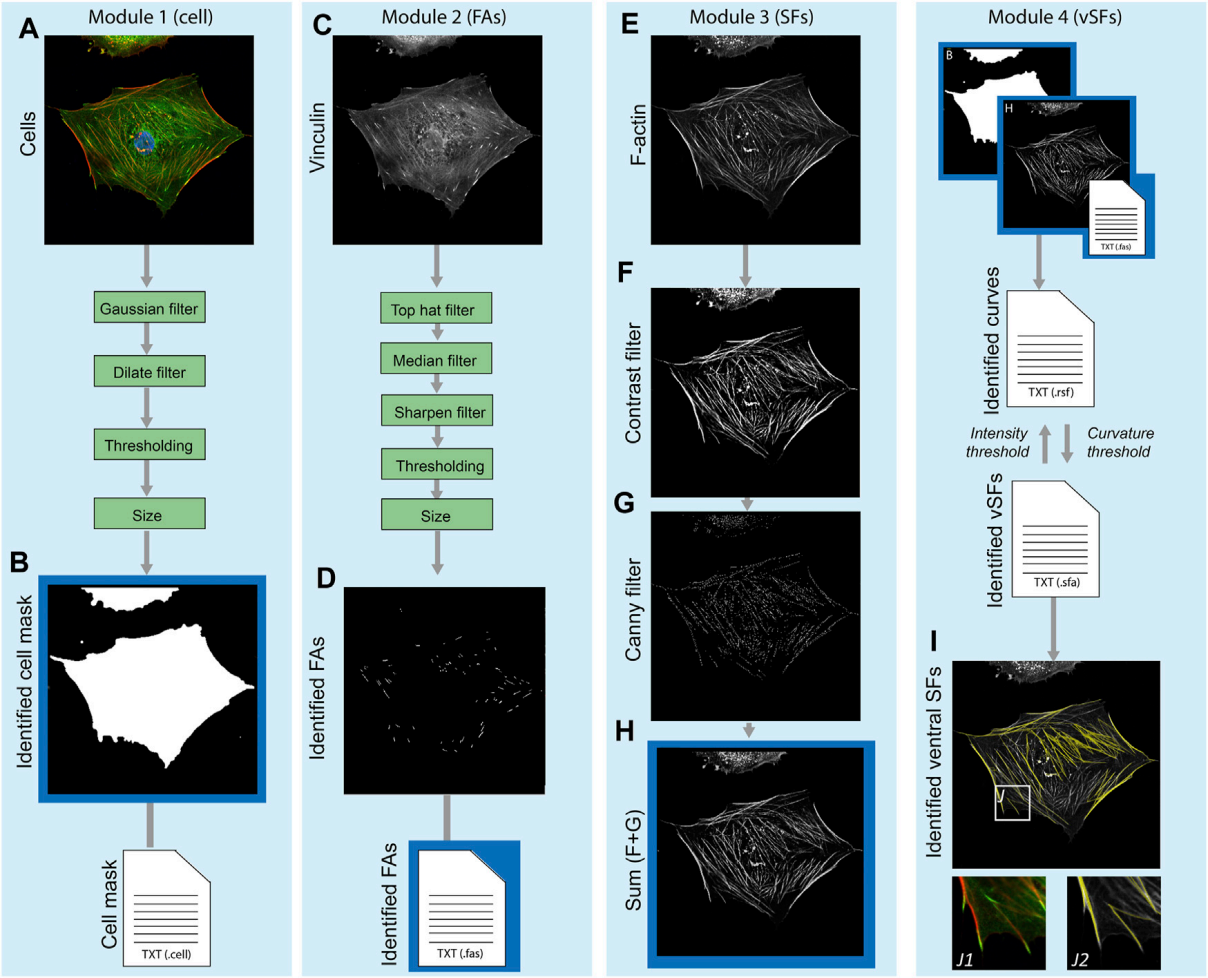
Schematic overview of the algorithm construction and working principle of SFAlab. **(A, B)** The raw data serving as input for SFAlab **(A)** and the mask created by the algorithm **(B)**. **(C, D)** The raw vinculin image **(C)** and the preprocessed FA image **(D). (E–H)** For the raw SF image **(E)**, subsequent contrast filtering **(F)** and edge detection **(G)** was used for image preprocessing, resulting in image **(H)**. Next, the identified cell mask, the spatial properties of the FAs, and the preprocessed SF image are used as input for the curve fitting procedure to identify the ventral SFs **(I)**. **(I)** Overlap between the raw SF channel (grey) and the identified SFs (yellow) with subfigures J1 and J2, indicating magnified images of **(J1)** an overlap of the vinculin and F-actin channel, and **(J2)** an overlap of raw SF channel and the identified SFs. Green boxes indicate the filtering techniques used for image preprocessing and the blue boxes show the different SFAlab modules. Four result files (text files) are generated containing the morphological and spatial characteristics of the mask, FAs, fitted curves, and identified ventral SFs.

The main principles (core functions) underlying the SFAlab algorithm are 1) fitting shapes to objects in the fluorescent images and extracting their spatial properties, and 2) fitting curves between two objects in the fluorescent images using maximum mean intensity. Besides these core functions, SFAlab contains multiple extended analysis functions that enable preprocessing of raw image files, and then initiate the appropriate core function for analysis. SFAlab can be divided into four modules: 1) the mask module for cell masking, 2) the FA module for shape fitting and the extraction of the properties of the identified objects, 3) the SF module, for curve fitting between two FAs in the same mask to identify ventral SFs and the extraction of their properties, and 4) the SF evaluation module, which allows the user to evaluate the SF output and extract and visualize the valid curves. The users can choose to either perform the entire analysis or only specific modules. A schematic algorithm construction and the working principle of SFAlab are visualized in Figure 1.

Within the first module, a binary cell mask is defined to determine properties of the identified FAs *per cell* and restrict the curve fitting procedure to find SFs only between FAs within a certain cell, and not between different cells. As input, the mask module uses a composite image of the FA and the SF channel (Figure 1A), normalizes the individual images to have a maximum intensity of 1, and sums up the individual channels, again having a maximum intensity of 1. Next, image dilation, Gaussian filtering, and binarization are performed. Finally, a size filter and border check allow the user to select the cell of interest for further analysis. The output is a binary image of the cell mask (Figure 1B) and a corresponding text file with the properties of the identified cell mask (e.g., area, location, orientation, *etc.*).

Since SFAlab uses the locations of FAs as the starting points to search for SFs, in Module 2 the FAs within the mask are identified using a set of image-filtering techniques to remove noise and any detectable signal from soluble vinculin from the raw FA images (Figure 1C). Since the method of feature extraction, together with the method of image pre-processing of the FA detection, are based on previously published work from our group, we refer to de Vries et al. (De Vries et al., 2014) and Buskermolen et al. (Buskermolen et al., 2018) for detailed explanations, respectively. After image preprocessing, FAs were segmented, their locations were identified, and several morphological features (e.g., area, aspect ratio) were quantified (Figure 1D). The output of the FA module is a text file with the morphological and spatial characteristics of all the identified FAs.

In the third module, image pre-processing of the SF image is performed, with the aim to emphasize the structural features of the SFs to enable SF identification (Figure 1E). The raw SF image is subsequently subjected to contrast stretching and to a Canny filter, which is an edge filtering technique (Ding and Goshtasby, 2001) (Figures 1F,G). The result of the pre-processing is the sum of the raw image, the contrast stretched image, and the edge filtered image (Figure 1H). After pre-processing of the SF image, the curve fitting procedure is performed to identify the ventral SFs. For the curve fitting, more specifically the fitting of a quadratic parabola of arbitrary orientation, the centroids of two FAs (FA_i_ and FA_j_) are superimposed on the preprocessed grey scale SF image, and the polynomial between those two points is fitted that has the largest mean intensity on the input image.

Lastly, in module four, the user can 1) visually inspect if SFAlab correctly fitted curves representing the ventral SFs, and 2) evaluate the mean arithmetic and geometric intensities, relative length and maximum (unsigned) curvature of all the fitted curves obtained. Since the parabola with the largest mean intensity may not resemble a ventral SF that is biologically reasonable, SFAlab implements a penalty function *p*) to constrain the curve fitting procedure:

$$p=\left\{\begin{array}{c} 1\;for\;\chi <t\\ e^{A\left(\chi -t\right)}\;for\;\chi \geq t \end{array}\right.$$ (1)

There are three penalty functions for three different curve variables (*χ*′s) with corresponding thresholds (*t*’s). First, the penalty function discourages the fitting algorithm to select parabolas that are rewinding (when a curve shoots beyond its target and loops back to one of the end points), where the variable *χ* is the amount of overshoot and the threshold is zero (hardcore). Besides curves that are rewinding, there is also a penalty function for curve length and for maximum curvature, of which both thresholds can be defined by the user. All three penalty functions share the same amplitude *A*, which can be set by the user as well.

When any of the detected curve properties passes a threshold defined in the curve penalty function, a penalty is added to the mean intensity of the curve (a multiplication factor), lowering its mean intensity, and discouraging the chance of this curve being identified as the curve with the highest mean intensity. When an attempted curve suffers from passing multiple user defined thresholds, the penalties on that curve accumulate.

Besides visual inspection and evaluation of the properties of the identified ventral SFs, module four also contains a feature to visualize the obtained ventral SFs over a background of choice (white, black, the raw SF image, or the preprocessed SF image) (Figure 1I).

### 2.3 Substrate preparations

Glass-bottom 12-well plates (MatTek, Ashland, United States) and microscope glass slides were treated with a 14:1:1 solution of absolute ethanol (VWR international, Amsterdam, the Netherlands)/acetic acid (Merck, Amsterdam, the Netherlands)/bind-silane (Sigma-Aldrich, Amsterdam, the Netherlands). After 1 h incubation, glass plates and slides were washed in ethanol and dried with nitrogen. PAA gels with a Young’s modulus of 12 kPa were prepared using a solution containing 93.8 μL acrylamide (40% w/v, Bio-Rad, Veenendaal, the Netherlands), 25 μL bis-acrylamide (2% w/v, Bio-Rad, Veenendaal, the Netherlands), 5 μL beads (ThermoFisher, Waltham, United States), 2.5 μL ammonium persulfate (10% w/v, Bio-Rad, Veenendaal, the Netherlands), and 0.25 μL TEMED (Merck, Amsterdam, the Netherlands) 373.5 μL in PBS. For traction force microscopy (TFM) purposes, 0.2 µm fluorescent beads were added to measure cellular traction forces while for immunofluorescence staining (IF) purposes, 0.2 µm non-fluorescent beads were added to keep the culture condition similar. Gels were prepared by pipetting 10 µL of PAA gel solution on the treated glass-bottom 12-well plate (TFM) or microscopy glass slide (IF). After pipetting, the PAA gel solution droplet was immediately covered with a Ø12 mm coverslip (ThermoFisher, Waltham, United States) to create flat PAA gels. PAA gels were left to polymerize for 1 h and coverslips were gently removed in PBS. Gel stiffness was verified (13.4 ± 2.0 kPa, n = 24 gels with 5 measurements per gel) using nano-indentation (Piuma Nano-indenter, Optics11, Amsterdam, the Netherlands). PAA gels were incubated with 1 mg/mL Sulfo-SANPAH (ThermoFisher, Waltham, United States) under 365 nm wavelength ultraviolet light for 5 min (Analytik Jena, Jena, Germany). After incubation, gels were washed extensively with sterile PBS before coating with 50 μg/mL rat-tail collagen type I (Corning, Amsterdam, the Netherlands) overnight at 4 °C. Collagen-coated PAA gels were washed twice in PBS, incubated with medium for 20 min and seeded with 750 cells (in 50 µL culture medium) per gel. When cells were attached after 4 h, medium was added. For TFM experiments, cells were cultured for 12 h after cell seeding on the PAA gels.

### 2.4 Cardiac fibroblast culture and Y-27632 treatment

Human epicardial-derived cardiac fibroblasts (cFBs) were kindly provided by Prof. M.J.Th.H. Goumans (Leiden University Medical Centre, the Netherlands). cFBs were isolated from human fetal cardiac tissue collected with informed consent and anonymously from elective abortion material of fetuses with a gestational age between 10 and 20 weeks. This research was carried out according to the official guidelines of the Leiden University Medical Center and approved by the local Medical Ethics Committee (number P08.087). cFBs were cultured in high-glucose Dulbecco’s modified Eagle’s medium (Invitrogen, Breda, the Netherlands) supplemented with 10% fetal bovine serum (Greiner Bio-one, Alphen aan den Rijn, the Netherlands) and 1% Penicillin Streptomycin (Gibco, Bleiswijk, the Netherlands). The cFBs were cultured in flasks coated with 0.1% gelatin from porcine skin in PBS (Sigma-Aldrich, Amsterdam, the Netherlands) and passaged at 80% confluency until they were seeded on the PAA substrates. For Y-27632 experiments, medium with 10 μM Y-27632 (StemCell Technologies, Vancouver, Canada) was added to the cells for 12 h before fixation for immunofluorescence staining.

### 2.5 Immunofluorescence staining

The cFBs cultured on the PAA gels for 24 h were washed with PBS thrice before and after fixation, and fixed in 3.7% formaldehyde (Merck, Darmstadt, Germany) for 15 min. The cells were permeabilized with 0.5% Triton-X-100 (Merck, Darmstadt, Germany) in PBS for 10 min and blocked for non-specific antibody binding with 10% horse serum (Sigma-Aldrich) in PBS for 40 min. The cells were incubated overnight with the primary antibodies at 4 °C in PBS with 1% horse serum. The cells were washed 6 times with PBS for 5 min before incubating with the secondary antibodies in PBS and phalloidin for 1.5 h. All used primary and secondary antibodies and dyes are listed in Supplementary Table S1. The cells were washed 2 times with PBS before incubating with DAPI in PBS for 5 min to visualize the cell nuclei. Finally, cells were washed 4 times with PBS for 5 min, and thereafter coverslips were mounted on top of the substrates with Mowiol (Merck, Darmstadt, Germany). Samples were stored protected from light at 4 °C until visualization. All images obtained were single confocal slices made at the basal side of the cells to minimize signal from the apical cell side to contribute to the SFs in the image. All images were acquired using a laser confocal microscope (Leica TCS SP8X) with a 63x/1.4 oil immersion objective at 100 Hz with 4x line averaging and a resolution of 2048 × 2048 pixels.

### 2.6 Traction force microscopy

Since ventral SFs are connected to the ECM at both ends, they are believed to be the main contributors to cellular traction forces (CTFs) (Soiné et al., 2015). To investigate this, we quantified CTFs live in cells while tampering with the formation of ventral SFs using Rho-associated protein kinase (ROCK) inhibitor Y-27632. Similar to the cells on PAA gels for immunofluorescence staining, medium with 10 μM Y-27632 was added to the cells for 12 h before starting CTF measurements. Before starting live cell and bead tracking, cells were washed in PBS and medium was added. Cells and beads were imaged using a Leica DMi8 microscope equipped with temperature and CO_2_ control (Leica, Wetzlar, Germany). Timelapse imaging was performed for 12 h, and images were acquired with a time interval of 30 min consisting of a phase contrast image for cell visualization and a fluorescence image for bead tracking. After finishing the timelapse, cells were removed by adding 5% SDS and a reference image was acquired. Using the reference image, images acquired over the timelapse were prepared for traction analysis by cropping and aligning images. Bead displacements between any experimental timepoint and the reference image were computed through a custom algorithm based on Particle Image Velocimetry Lab (PIVlab) (Thielicke and Sonntag, 2021) using an interrogation window of 32 × 32 pixels and a 50% overlap. CTFs were computed using a Fourier transform algorithm for elastic hydrogel substrates with a finite thickness (Trepat et al., 2009). Individual cells were selected and a mask surrounding the cell was manually prepared for every timepoint. The traction force magnitude of a cell in each timepoint was determined by calculating the median value of all the traction magnitude values within the mask of the cell. The traction magnitude of a cell over time was calculated as the mean of calculated median values of each timepoint. For all CTF calculations, MATLAB Version: 9.13.0.2,049,777 (R2022b) was used.

### 2.7 Statistics

All data are presented as mean ± SEM. For two group comparisons, a Student’s t-test was used at 95% confidence interval after normality and equal variances were checked with Shapiro-Wilk test and Brown-Forsythe test, respectively. For three group comparisons, a one-way analysis of variance (ANOVA) was used at 95% confidence interval after normality and equal variances were checked with Shapiro-Wilk test and Brown-Forsythe test, respectively. For all comparisons, *p* < 0.05 was considered statistically significant.

## 3 Results

### 3.1 Validation of SFAlab

**FIGURE 2:**
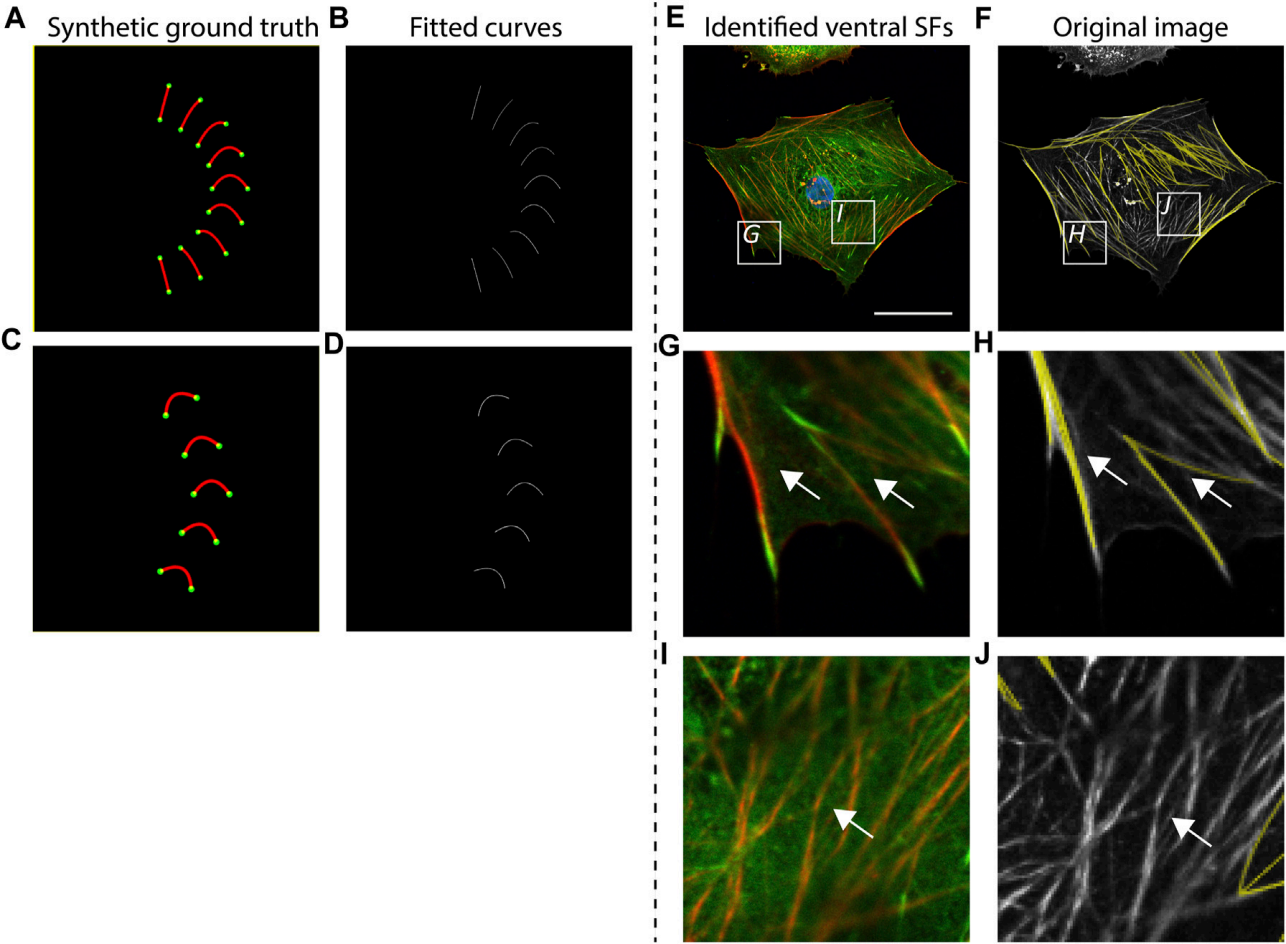
Accuracy of curve fitting procedure and qualitative validation of SFAlab. **(A, C)** Synthetic ground truth images containing almost straight lines and curves, mimicking stress fiber (SF) shapes found in cells. **(B, D)** Rendered SF image indicating accurate detection of the ground truth. **(E, G, I)** Representative raw data showing a cardiac fibroblast at a cellular level and two zoomed images depicting subcellular locations where ventral SFs are expressed **(G)** or not expressed **(I)** (nucleus = blue, vinculin = green, F-actin = red). **(F,H J)** The curve fitting procedure of SFAlab can identify the ventral SFs from the raw image (raw image = grey, identified curves = yellow) and does not detect SFs that do not originate from and terminate in a focal adhesion (white arrows). Scale bar = 50 μm.

To validate the accuracy of SFAlab in identifying ventral SF, we created artificial ventral SFs images (as synthetic ground truth) and analyzed these images using SFAlab. Besides validation against a ground truth, we also qualitatively assessed accurate detection of ventral SFs by visual correlation of the rendered SFs from SFAlab with a representative raw immunofluorescence cell image (Figure 2).

First, we created curved SFs and analyzed these structures using SFAlab (Figures 2A,B). We found that SFAlab was able to identify these structures and that the output of SFAlab contained the same number of FAs, SFs, and SFs/FA as presented in the input figure. Moreover, in contrast to the existing tools available to quantify ventral SFs to date (Elosegui-Artola et al., 2014), SFAlab allows the user to quantify ventral SFs in cells of arbitrary orientations, since this is necessary for comparison of the same ventral SF over time (e.g., during timelapse imaging). In brief, we parametrized the curve that goes into the intensity optimization algorithm with distance *d*, angle *θ*, and *a* for a parabola *y = ax* ^*2*^ *+ bx + c*. This allows us to get a curve with arbitrary curvature and skew from a second-order polynomial. So for each pair of FAs we only use their distance d and not their actual pixel coordinates to generate curves with various values for *a* and *θ* for that distance *d* (Supplementary Figure S1), These curves are then rotated and translated to the actual pixel coordinates of the FAs in the image To validate this, we artificially created five curves with identical shape descriptors except for the orientation and assessed how well the SFAlab algorithm identified these curves (Figures 2C,D). Like the curves and lines with different shape descriptors from Figure 2A, the SFAlab output showed good agreement with respect to the original image, indicating the suitability of SFAlab to analyze fibers of arbitrary orientation.

To investigate the performance of the algorithm to detect ventral SFs in cells, we determined the correlation between visually detected ventral SFs from an immunofluorescence image of a single confocal slice at the basal side of a cell and the identified ventral SFs from SFAlab at a cellular (Figures 2E,F) and subcellular scale (Figures 2G–J). Visual comparison on both scales shows good agreement between the ventral SFs from the raw image and the curves fitted by SFAlab (Figures 2G,H, white arrows). Moreover, SFAlab ignores actin fibers not originating from nor terminating at a FA (Figures 2I,J, white arrow).

### 3.2 Accuracy of SFAlab in the presence of image corruptions

**FIGURE 3:**
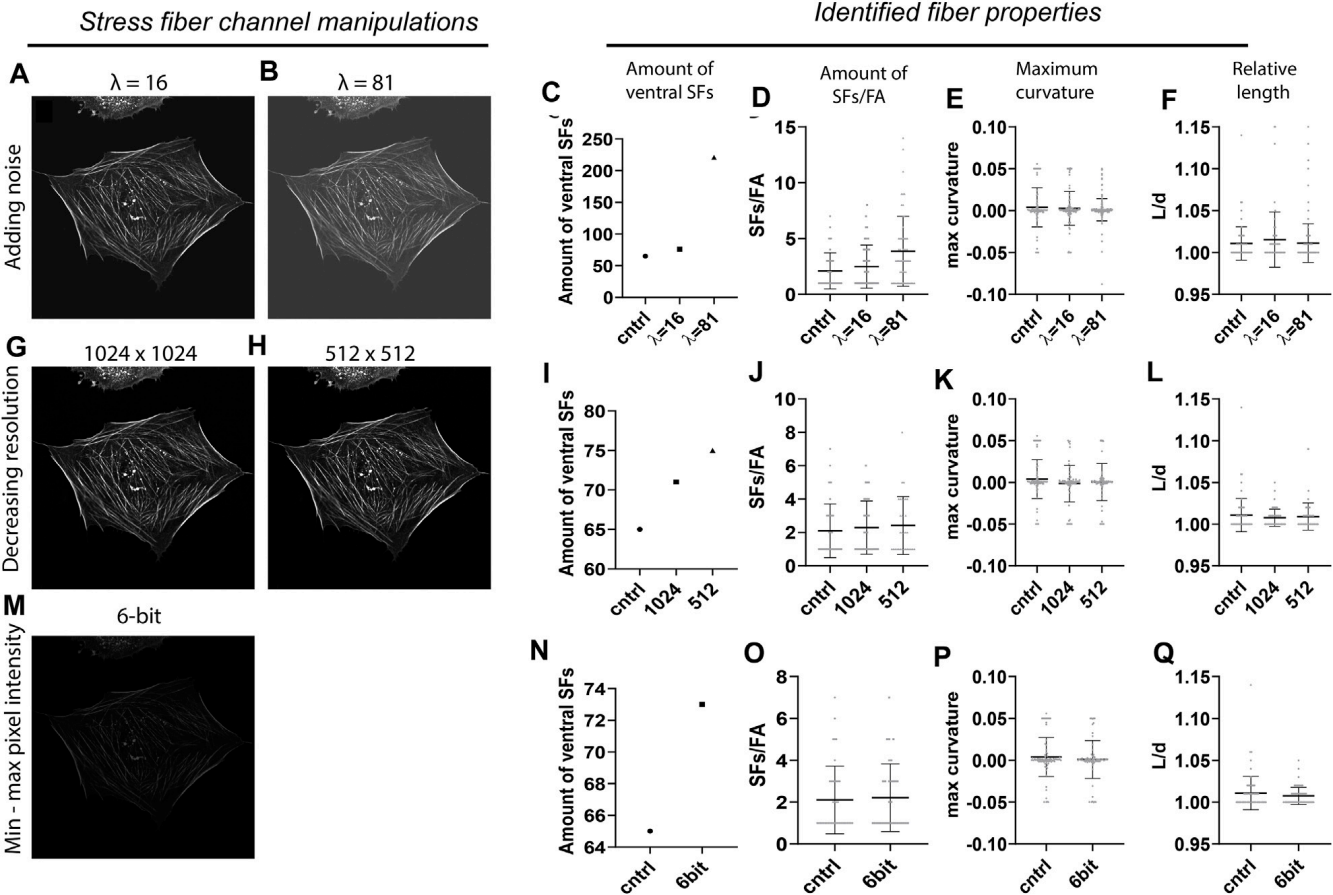
Accuracy of the SFAlab algorithm upon image manipulations. **(A,B)** Representative preprocessed SF image after adding Poisson noise. **(C-F)** Properties of the identified ventral SFs upon noise addition. **(G-H)** Representative preprocessed SF image after decreasing resolution. **(I-L)** Properties of the identified ventral SFs decreasing resolution. **(M)** Representative preprocessed SF image after decreasing bit depth. **(N-Q)** Properties of the identified ventral SFs upon decreasing bit depth. The accuracy of the SFAlab algorithm upon image manipulations was assessed for five more cells and this data can be found in Supplementary Figure S2.

To assess the accuracy of SFAlab, we tested different imaging conditions by intentionally corrupting the original SF image with artifacts that are often found in fluorescence micrographs as a result of non-ideal imaging settings. The control image contained no artificially added Poisson noise (λ = 0), had an image resolution of 2048 × 2048 pixels by pixel, and was 8-bit. In short, the following image corruptions were implemented: 1) Poisson noise was added for two arbitrary values (*λ* = 16, 81) to simulate imaging imperfections due to noise, 2) image resolution was decreased to 1,024 × 1,024 and 512 × 512 pixels to simulate different microscopy settings, and 3) the SF image was converted to a 6-bit image to scale the dynamic range to simulate differences in bit depth. The results of the effect of these image condition manipulations on the amount of identified ventral SFs, the number of SFs/FA, and the maximum curvature and relative length can be found in Figure 3. The relative length is defined as the length of the curve L) divided by the distance d) between the two FAs (L/d).

When the original SF image was noise corrupted (Figures 3A,B), the number of detected SFs and with that the amount of SFs/FA increased with increasing *λ* (Figures 3C,D). Specifically, with *λ* = 16, the number of SFs increased to 76 and the amount of SFs/FA to 2.4 ± 1.9 as opposed to the control image with 65 identified SFs and 2.1 ± 1.6 SFs/FA. When *λ* was increased to 81, the number of SFs increased to 221, with a significant increase in the amount of SFs/FA to 3.9 ± 3.1 as opposed to the control (*p* = 0.002) or to the *λ* = 16 condition (*p* = 0.02) (Figure 3D). For the relative length and the maximum curvature, no effect of noise corruption was found (Figures 3E,F). It should be noted that the increase in number of SFs was not an effect of an increase in signal intensity, since the intensity threshold for accepting curves as SFs was proportionally adjusted to the amount of intensity added by the noise. Moreover, when the intensity was adjusted to detect the same number of SFs from each image, some identified SFs in the *λ* = 16 and 81 image differed from the control, indicating a loss of accuracy in ventral SF detection with increasing noise. When the resolution or the dynamic pixel intensity range was decreased, only a slight increase in the number of SFs was found, with no significant effect on the amount of SFs/FA or the maximum curvature of relative length of these SFs (Figures 3I-L, Figures 3N-Q).

Together, these results indicate that ventral SF identification by SFAlab is most susceptible to noise corruption and hardly affected by differences in resolution or bit depth. This highlights the applicability of SFAlab for multiple imaging conditions and the importance of minimizing noise corruption during image acquisition when using SFAlab.

### 3.3 Robustness of SFAlab towards user subjectivity

Since image preprocessing can be prone to user subjectivity, we assessed the robustness of our algorithm by testing how variations in the preprocessing parameters of the SF module affect the results obtained by SFAlab. During preprocessing of the SF image three parameters can be set: 1) the image intensity values to be mapped (contrast stretching), 2) the thresholds for the canny filter (edge detection), and 3) the weight of the contrast stretched image and the edge detection image in determining the final preprocessed SF image. In the control image the pixels between the lowest 10% and highest 50% were mapped, the threshold values for the canny filter were set to 0.005 and 0.3, and the weight of the contrast stretched image *versus* the edge detection image was 3 to 1. The values of the SF preprocessing parameters that led to the most accurate overlap between rendered ventral SFs and ventral SFs found visually were chosen as control values.

**FIGURE 4:**
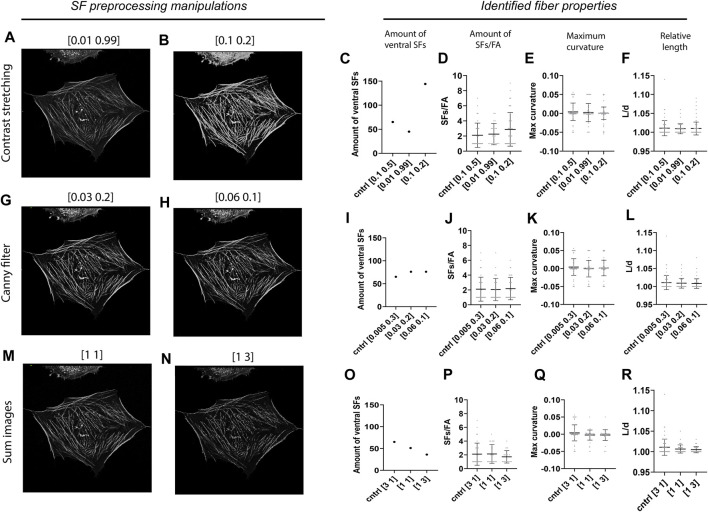
Robustness of the SFAlab algorithm. **(A-B)** Representative preprocessed SF image after contrast stretching for different values. **(C-F)** Properties of the identified ventral SFs upon manipulation of the threshold values for contrast stretching. **(G-H)** Representative preprocessed SF image after applying edge detection for different values. **(I-L)** Properties of the identified ventral SFs upon manipulation of the threshold values for edge detection. **(M-N)** Representative preprocessed SF image after altering the weight of the contrast stretched and the edge detection image. **O-R)** Properties of the identified ventral SFs upon adapting the weight of the contrast stretched and the edge detection image in determining the final SF image. The robustness of the SFAlab algorithm was assessed for five more cells and this data can be found in Supplementary Figure S3.

When altering the upper- and lower-pixel value limits over which the image was to be stretched, a clear increase was found in the number of ventral SFs detected when the difference in intensity between SFs in the raw SF image was decreased, together with a slight increase in the amount of SFs/FA (Figures 4A–D). Specifically, when only 2% of the pixel values was excluded, as opposed to 60% in the control sample, SFAlab detected 45 SFs compared to the 65 SFs identified for the control. Moreover, when 90% of the pixel values were excluded, the algorithm detected 144 SFs. The increase in number of SFs detected when more contrast stretching was applied can be explained as follows: because SFAlab fits curves based on the mean intensity, a low intensity SF that is in close vicinity to a high intensity SF will not be identified as the highest mean intensity projection and therefore not be detected. However, when significant contrast stretching is applied, the intensity difference between those two fibers becomes less, allowing SFAlab to fit curves on both SFs. For maximum curvature and relative length, no differences were observed upon contrast stretching (Figures 4E,F).

**FIGURE 5:**
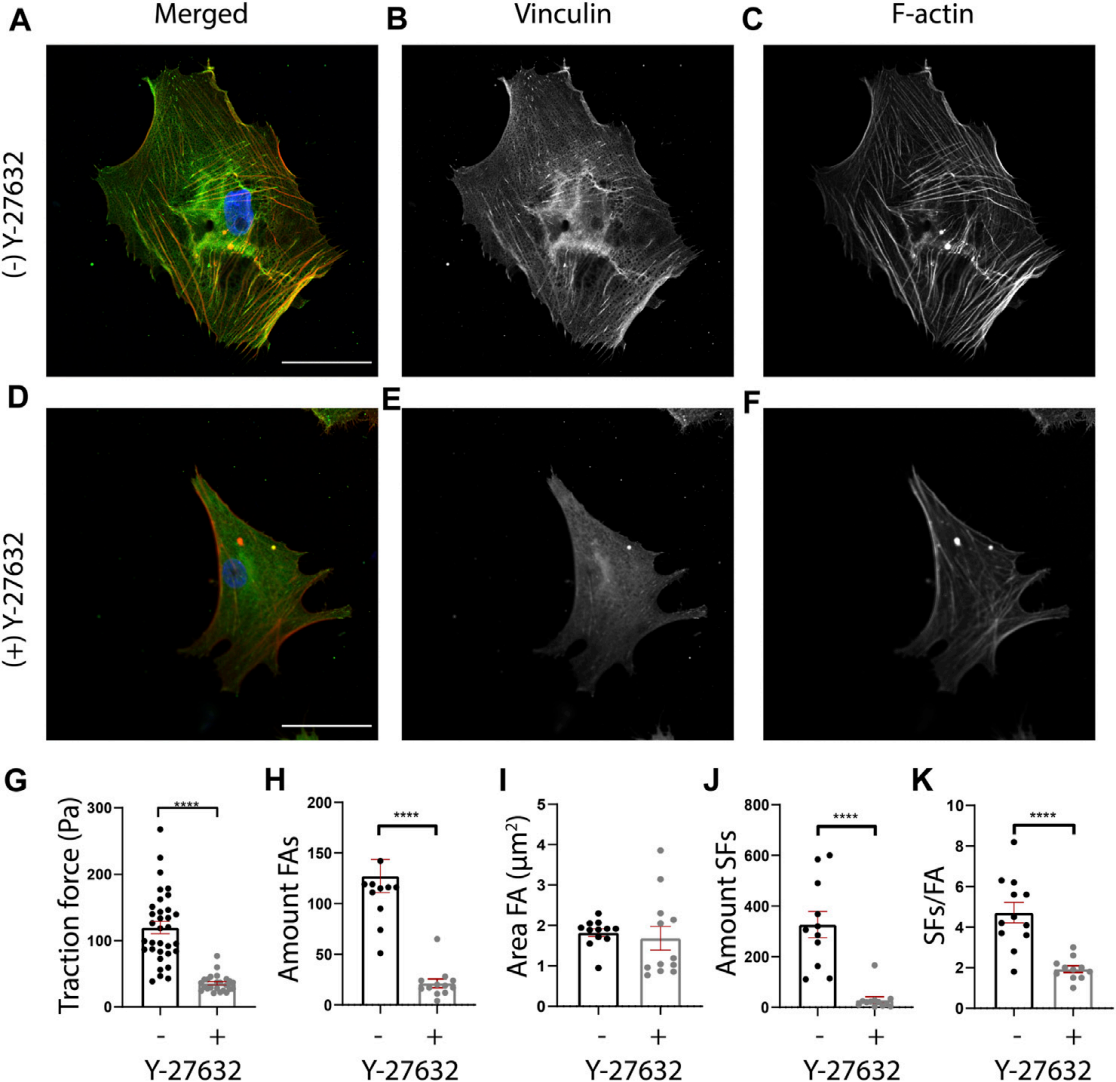
Y-27632 affects cellular morphology, cellular traction forces, and FA and SF presence in human cardiac fibroblasts (cFBs). **(A-F)** Representative fluorescent images of cFBs after 24 h of culture without Y-27632 **(A-C)** or with Y-27632 **(D-F)**. Scale bar = 50 μm. **(G)** Quantification of cellular mean traction forces measured on 12 kPa polyacrylamide gels ((−) Y-27632, n = 32 cells (+) Y-27632, n = 29 cells). **(H)** Quantification of the number of FAs (number of cells (−) Y-27632, n = 12 (+) Y-27632, n = 12). **(I)** Quantification of the FA area (number of cells (−) Y-27632, n = 12 (+) Y-27632, n = 12). **(J)** Quantification of the number of ventral SFs (number of cells (−) Y-27632, n = 12 (+) Y-27632, n = 12). **(K)** Quantification of the number of ventral SFs per number of FAs.

With increased thresholds for the edge detection filter (Figures 4G,H), only a slight increase was observed for the number of identified SFs, and no difference was found for the number of SFs/FAs (Figures 5I,J). Similar to contrast stretching, maximum curvature and relative length of the identified ventral SFs were not affected by differences in thresholds for edge detection (Figures 4K,L).

Lastly, when increasing the weight of the edge detection image in creating the final preprocessed SF image, SFAlab detected fewer SFs and SFs/FAs (Figures 4O,P). Specifically, choosing equal weights for the contrast stretched image and the edge detection image resulted in 51 detected SFs, whereas giving the edge detection image a three times higher weight than the contrast stretched image resulted in 36 identified SFs. Additionally, whereas no significant differences were detected in the amount of SFs/FA between the control image and when both images had similar weights, a significant decrease was found when the edge detection image had a three times higher weight than the contrast stretched image.

Together, these results indicate the dependency of the properties of the identified SFs on the subjectivity of the user and highlight the importance of contrast stretching and the weight of image *A* and *E* in determining the number of identified ventral SFs and the amount of SFs/FA.

### 3.4 SFAlab shows that Y-27632 treatment decreases amount of ventral SFs

Having validated that ventral SFs could be identified by SFAlab, we next used SFAlab to investigate the effect of the RhoA pathway on ventral SF formation, by treating human cFBs with Y-27632, a selective inhibitor of Rho-associated kinase (ROCK). First, to assess the effectiveness of Y-27632 on cFBs, we qualitatively assessed FA and SF expression (Figures 5A–G) and determined cellular traction forces (CTFs), as literature shows that Y-27632 treated cells exhibit reduced levels of internal stress (Beningo et al., 2006). In order to keep similar experimental conditions, all cells analyzed were cultured on 12 kPa PAA gels. Whereas the untreated cFBs showed clear FA and SF expression (Figures 5A–C), the presence of FAs was decreased and the SF formation was disrupted in the cells treated with Y-27632, which is in accordance with what is shown in literature (Iwamoto et al., 2000; Polio et al., 2014) (Figures 5D–F). Moreover, determining the average CTFs revealed that cells treated with Y-27632 show impaired cell contractility (*p* < 0.01) (Figure 5G). Together, these results confirm the effectiveness of Y-27632 in impairment of SF assembly and reducing CTFs.

Having confirmed that Y-27632 treatment affected the CTFs of cFBs, we next investigated the effect of RhoA inhibition on ventral SF formation using SFAlab. Since ventral SFs originate and terminate in FAs, we first determined the FA expression in cells treated with and without Y-27632. In line with previous work (Lavelin et al., 2013; Stricker et al., 2013; Buskermolen et al., 2018), we found a significant decrease in the number of FAs present in the treated cells as opposed to the untreated cells (*p* < 0.01) (Figure 5H). In addition, a decrease in FA area was observed, albeit not statistically significant (Figure 5I; *p* = 0.51). Corresponding with the qualitative analysis, both the number of SFs and the amount of SFs/FA decreased significantly in Y-27632 treated cells as opposed to the untreated control (Figures 5J,K). Moreover, we found no difference in the maximum curvature but a significant increase in the relative length of the ventral SFs detected upon Y-27632 treatment as opposed to the untreated control (Supplementary Figure S4). Together, these results indicate a decrease in FA expression, concurrent with a decrease in ventral SF formation upon inhibition of RhoA associated SF formation. Also, this data demonstrates the usefulness of SFAlab for obtaining quantitative insights to potentially aid in studying the role of ventral SFs in mechanoreciprocity.

## 4 Discussion

An easy-to-use and reliable method to quantify ventral SFs in adherent cells in 2D would be of significant benefit to unravel mechanisms underlying ventral SF formation and to better understand the role of ventral SFs in mechanoreciprocity. Quantification of actin SFs using image analysis-based approaches has been successfully used before (Rogge et al., 2017; Zhang et al., 2017; Zonderland et al., 2019; Nowak et al., 2020), but quantification of the subset of ventral SFs remained challenging so far. Ventral SFs are of importance in mediating mechanosensing and mechanoresponsive because of their direct connection to the extracellular environment and the strong traction forces they can produce (Soiné et al., 2015). In this study we present an image-based computational framework, called SFAlab, to identify and quantify ventral SFs from microscopic fluorescence images of cells. Whereas detection and quantification of SFs in general is useful, being able to quantify a subset of SFs, the ventral SFs, is especially valuable to learn about the function of these structures. Besides the number of ventral SFs, SFAlab also enables detection of the amount of SFs per FA and gives spatial information about the locations of the detected ventral SFs, providing important insights for mechanobiological studies.

Validation of SFAlab indicated good agreement with the ventral SFs detected *via* visual inspection of fluorescence microscopy images and with the imposed ventral SFs in a synthetic ground truth. We note that local artefacts or imaging corruptions might hamper SFAlab to accurately detect ventral SFs in an image, indicating the relevance to visually validate the identified ventral SFs (and their shape descriptors) using the built-in evaluation tool of SFAlab. Analysis of the accuracy and robustness of SFAlab revealed that the detection of ventral SFs is most affected by noise corruption and by contrast stretching during preprocessing of the raw SF image. This indicates the importance of considering the degree of noise and maintaining a similar amount of noise between images when analyzing images with SFAlab. To our knowledge, the accuracy and robustness of ventral SFs identification with respect to typically occurring imaging imperfections and user subjectivity has not been assessed before. Yet, Eltoner et al. also acknowledges a decrease in accuracy when using their FilamentSensor tool to detect SFs in fluorescence images with noise corruption (Eltzner et al., 2015). Besides considering noise, optimizing the right amount of contrast stretching and keeping this variable constant between experimental groups is of importance for accurate ventral SF detection and for comparing results between experimental groups. Especially when the raw image contains SFs with low mean pixel intensity in proximity of SFs with high mean pixel intensity, saturating a vast number of pixel intensity values is advised to also detect the low mean pixel intensity SFs. To simplify this optimization, the user can assess the effect of the amount of contrast stretching on ventral SF detection using the SF-evaluate function of SFAlab.

Human cFBs were treated with Y-27632 to compare ventral SF expression with respect to untreated cells. The output of SFAlab demonstrated the influence of the RhoA effector ROCK on the expression of ventral SFs, showing a decrease in ventral SF presence upon ROCK inhibition. These results were in line with our expectations since ventral SFs exert the strongest cellular traction forces of all the SF subtypes (Soiné et al., 2015; Narasimhan, 2022). However, an increase in relative length and no significant difference in maximum curvature of the detected ventral SFs contradict the outcome of two studies that investigate the effect of Y-27632 on the shape of peripheral arcs, showing a decrease in maximum curvature and relative length upon Y-27632 treatment (Bischofs et al., 2008; Labouesse et al., 2015). These opposing results might be explained by the fact that both studies investigate the shape descriptors of a few (confined) peripheral SFs, whereas we consider all ventral SFs present in a cell. Whereas the effect of Y-27632 on SFs in general in well documented (Svoboda et al., 2004; Gates et al., 2007; Katoh et al., 2007), our study is the first to our knowledge that quantitatively shows the effect of ROCK inhibition on ventral SFs specifically, making use of the feature of SFAlab that enables assessment of only ventral SFs. We demonstrated that Y-27632 treatment reduced the number of FAs and ventral SFs, and the amount of SFs/FA.

Besides quantification of the number of ventral SFs, SFAlab provides spatial information about the location of these ventral SFs, and about the amount of SFs/FA. Moreover, SFAlab allows the possibility to assess the orientation of these ventral SF to shed light on cell polarity, although this output parameter is not included in the present study. While not possible with the current study design because of FA and SF visualization in fixed cells, we envision a promising role of combining SFAlab and traction force microscopy with live-cell imaging of FAs and SFs, to provide spatiotemporal information about the role of ventral SFs in mechanosensing and mechanoresponsive. In fact, since the curve fitting procedure in SFAlab works independently of ventral SF orientation, it allows the identification and quantification of ventral SFs in actively migrating live cells. This way, the spatial information gathered from SFAlab could be linked to the spatial output of TFM, assessing the importance of ventral SFs and the number of SFs/FAs in generating local cellular traction forces.

We focus on the identification of ventral SFs in the present study, but SFAlab is built as an all-in-one toolbox that also enables the identification and quantification of the shape descriptors of nuclei, cells, and FAs. While several studies have reported separate algorithms for the quantification of nuclei, cells, and FAs (Buskermolen et al., 2018), it is advantageous to combine these quantifications in the same cells, using a single user-friendly graphical user interface and the same core functions for each analysis, making it possible to couple data from the different analysis functions. It should be noted that the results in the present study are focused on FA protein vinculin and the cytoskeletal protein F-actin, but also other FA proteins, such as integrins and zayin (Goldmann, 2012; Kuo, 2013), can be analyzed to detect ventral SFs using SFAlab. Moreover, SFAlab is not limited to analysis on single cells but it can quantify the expression of ventral SFs in multiple cells at once due to the masking procedure if the cells to be analyzed do not touch each other. Since SFAlab is Open Source the algorithm can be expanded to extend its usage, for example, with a function to quantify ventral SF width or cell polarity based on ventral SF orientation.

To conclude, we present a robust and accurate image analysis-based approach to quantify ventral SFs in adherent cells in 2D, which can be a valuable analytical tool to gain deeper knowledge of the mechanisms of ventral SF formation and of their role in mechanosensing and mechanoresponsive.

## Data Availability

The raw data supporting the conclusion of this article will be made available by the authors, without undue reservation.

## Supplementary material

SUPPLEMENTARY FIGURE S1
**(A, B)** Drawbacks of the formula *y = ax* ^*2*^ *+ bx + c*. **(A)** With polynomials *y = ax* ^*2*^ *+ bx + c* through focal adhesions *F*_*i*_(*x*_*i*_=1, *y*_*1*_ = 3) and *F*_*j*_ = 11, *y*_*j*_ = 12) (black dots), colour coded for various values for −0.1 ≤ *a* ≤0.1. This results in skewed profiles when *y*_*i*_ *≠ y*_*j*_ and the skew is fixed: if the skew had a different sign, the fit through the red point at (10,9) (dashed black line) would have looked like a mirror image of the blue line for *a* = −0.1 but we cannot fit that curve with these equations. **(B)** Polynomials *y = -0.15x* ^*2*^ *+ bx + c* through a focal adhesion of length 10 in different colour coded orientations: 75° ≤ *θ* ≤ 75°. The orientation of the skew depends on the sign of the angle, and the appreciable (symmetric) curvature for a = −0.15 degenerates to a straight line for large rotations away from the horizontal (−75°, 75°). **(C, D)** For a given distance *d* between two FAs, we can get a curve with arbitrary curvature and skew from a second-order polynomial by variation of the parameters *a* and *θ*. **(C)** The parameters *a* and *θ* allow you to select any part from a second-order polynomial for given *d* (black lines, *d* = $\frac{3}{4}$ ). **(D)** The selected curves (bold) are rotated and translated to the positions and image orientations of the FAs (black dots) for evaluation of the curves mean intensity. Note that an opposite skew is simply obtained by changing the sign of *θ* for given *d* and *a* (dashed curves).

SUPPLEMENTARY FIGURE S2 Accuracy of the SFAlab algorithm upon image manipulations. **(A–D)** Properties of the identified ventral SFs upon noise addition. **(E–H)** Properties of the identified ventral SFs after decreasing resolution. **(I-L)** Properties of the identified ventral SFs upon decreasing bit depth. N (number of cells analyzed) = 6.

SUPPLEMENTARY FIGURE S3 Robustness of the SFAlab algorithm. **(A-D)** Properties of the identified ventral SFs upon manipulation of the threshold values for contrast stretching. **(E-H)** Properties of the identified ventral SFs upon manipulation of the threshold values for edge detection. **(I-L)** Properties of the identified ventral SFs upon adapting the weight of the contrast stretched and the edge detection image in determining the final SF image. N (number of cells analyzed) = 6.

SUPPLEMENTARY FIGURE S4 Maximum curvature and relative length of ventral SFs detected in cells upon Y-27632 treatment as opposed to the untreated control cells (number of cells, (−) Y-27632, n = 12, (+) Y-27632, n = 12). * = *p* < 0.05.
