# Supplementary figures and images for: SFAlab: image-based quantification of mechano-active ventral actin stress fibers in adherent cells
Source: Front Cell Dev Biol. 2023 Sep 15;11:1267822. doi: 10.3389/fcell.2023.1267822 (PMC10540851; doi:10.3389/fcell.2023.1267822)

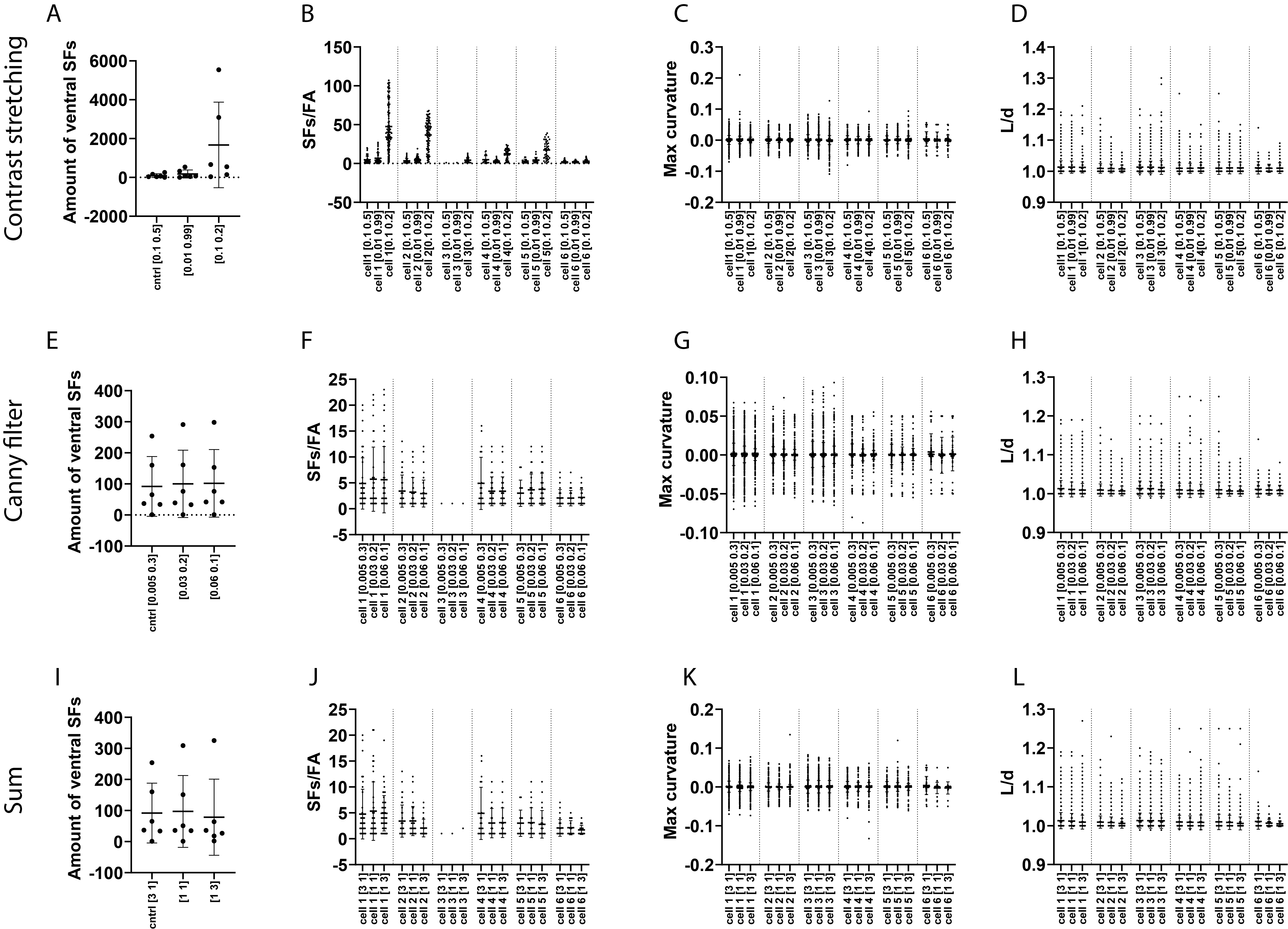

Supplement: Supplementary file 1 [file Image3.TIF]

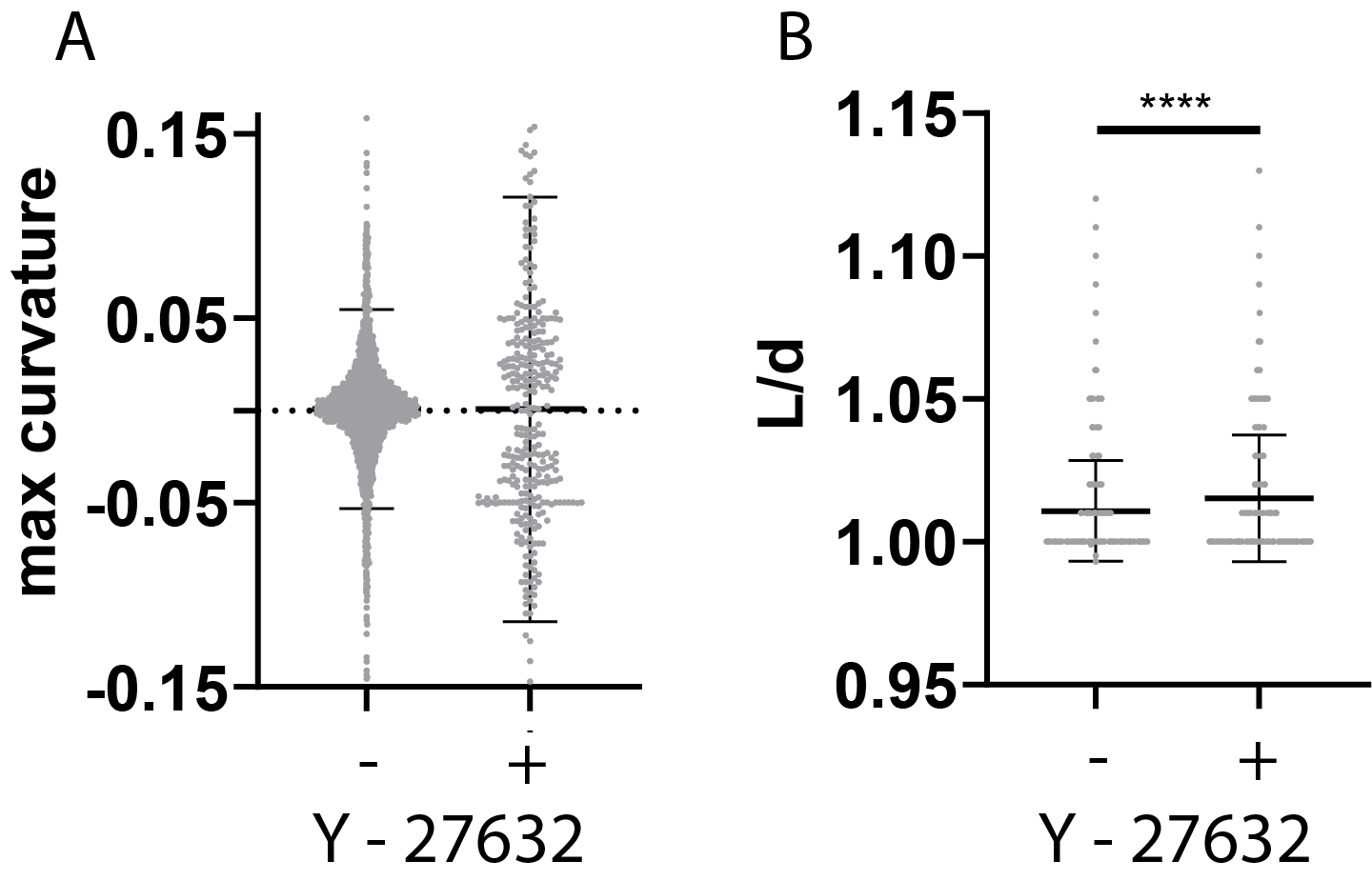

Supplement: Supplementary file 2 [file Image4.TIF]

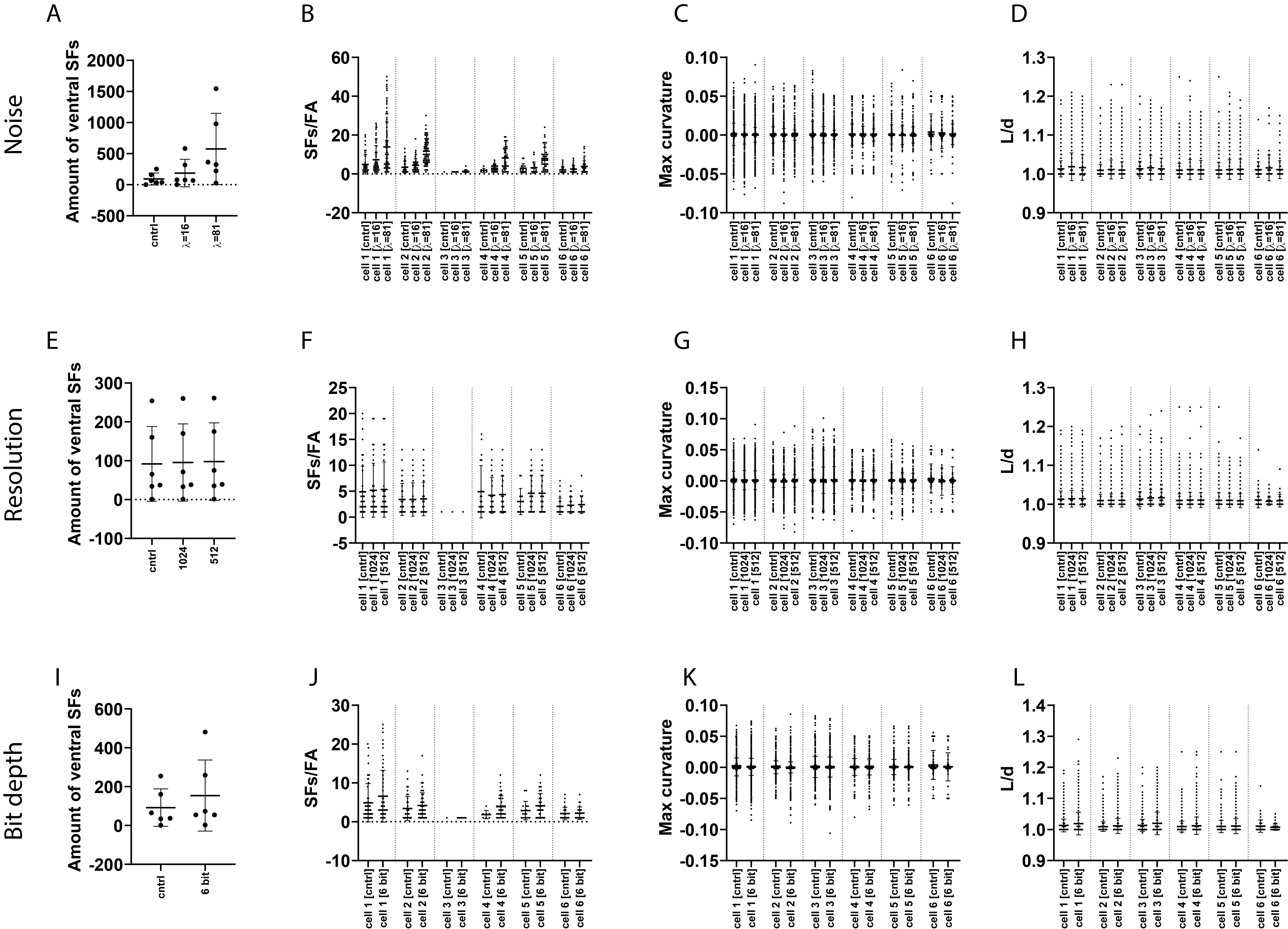

Supplement: Supplementary file 3 [file Image2.TIF]

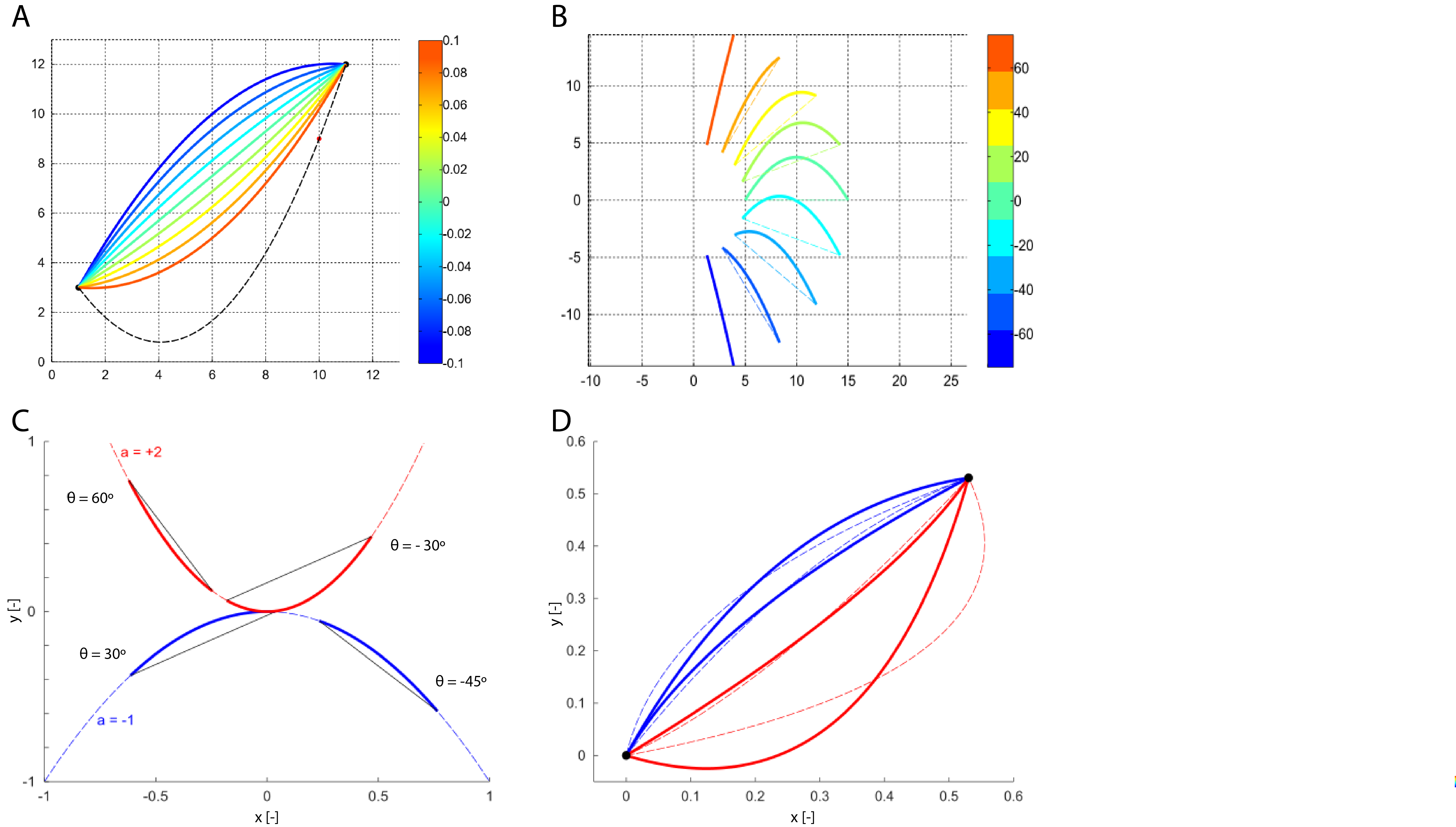

Supplement: Supplementary file 4 [file Image1.TIF]
